# Functional relevance for central cornea thickness-associated genetic variants by using integrative analyses

**DOI:** 10.1186/s13040-018-0179-3

**Published:** 2018-08-15

**Authors:** Jing Zhang, Dan Wu, Yiqin Dai, Jianjiang Xu

**Affiliations:** Department of Ophthalmology and Visual Science, Eye Institute, Eye & ENT Hospital, Shanghai Medical College of Fudan University, NHC Key Laboratory of myopia (Fudan University), Shanghai, China

**Keywords:** Central cornea thickness, GWAS, Integrative analyses, Regulatory variants

## Abstract

**Background:**

The genetic architecture underlying central cornea thickness (CCT) is far from understood. Most of the CCT-associated variants are located in the non-coding regions, raising the difficulty of following functional characterizations. Thus, integrative functional analyses on CCT-associated loci might benefit in overcoming these issues by prioritizing the hub genes that are located in the center of CCT genetic network.

**Methods:**

Integrative analyses including functional annotations, enrichment analysis, and protein-protein interaction analyses were performed on all reported CCT GWAS lead SNPs, together with their proxy variants. Functional annotations were conducted by CADD, GWAVA, and Eigen. Enrichment analyses for CCT-associated genes were performed using ToppGene suite. Protein-protein interaction network and gene co-expression analyses were performed by GeneMANIA.

**Results:**

Functional annotations prioritized eight genes (*ADAMSTS6, ARID5B, FOXO1, AKAP13, COL4A3, COL8A2, TBL1XR1,* and *KCMB2*) harboring SNPs with strong evidence of regulatory potential. It was also shown that CCT-associated genes were significantly enriched in collagen-related pathways and the phenotype of keratoconus, and some of them were found to be involved in one interaction network.

**Conclusion:**

This study revealed the hub genes that were located in the center of CCT genetic network and provided a new insight into the genetic regulation underlying CCT GWAS findings.

**Electronic supplementary material:**

The online version of this article (10.1186/s13040-018-0179-3) contains supplementary material, which is available to authorized users.

## Introduction

The cornea is a highly collagenous, transparent tissue through which light reaches the interior structures of the eye. Corneal thickness is closely related to corneal refractive power, which contributes to normal vision. Epidemiologic studies have shown that central cornea thickness (CCT) values differ among ethnic groups, with Europeans have higher CCT values than Africans, and Asians have a broad variation in CCT [1]. There have been growing evidences suggested that changes in CCT are closely related to ocular abnormalities. For example, reduced CCT or extreme thinner CCT has been observed in some rare ocular disorders, including brittle cornea syndrome, osteogenesis imperfect, and Ehlers-Danlos syndrome [2, 3]. Moreover, CCT has also been demonstrated as an important indicator for several ocular diseases with complex etiology. A thinner CCT has been demonstrated as an important feature of keratoconus and a risk factor for primary open-angle glaucoma (POAG) in patients with ocular hypertension [4, 5]. However, the contributing factors that influence CCT values remain elusive, and intensive exploration of such information may help in explaining how CCT values are defined, and also benefit in revealing the relationship between CCT and certain ocular diseases.

CCT has been shown as a highly heritable, normally distributed quantitative trait. A line of evidences from familial and twin studies indicated a strong genetic component underlying CCT, with heritability estimated as high as 95% [6, 7]. However, the genetic determinants of CCT remained unclear until the wide application of genome wide association studies (GWAS) in complex traits. With such approach, a number of CCT-associated loci have been identified in Europeans and Asians, including *ZNF469, FOXO1, LRRK1, IBTK,* and several collagen-related genes [8–14]. In a recently published meta-analysis of CCT on over 20,000 individuals in Europeans and Asians [9], 16 novel loci were identified, and some of them conferred relatively high risks for keratoconus and POAG, highlighting the potential involvement of CCT-associated genes underlying the pathogenesis of particular ocular diseases. However, quite few of these findings showed benefits in translational medicine for early diagnosis and treatment [15]. Moreover, it is also difficult to distinguish neutral CCT-associated variants from the pathogenic ones that actively participate in causing ocular abnormalities [1]. One of the underlying reasons is that most of the associated variants are located in the non-coding regions, raising the difficulty of following functional characterizations. However, it is possible that some variants might play critical regulatory roles yet to be found [16]. It is also reasonable to speculate that some variants that located in genes belonging to critical biological pathways are more likely to confer disease risk, rather than merely determining normal CCT variation. Therefore, there is an urgent need to explore molecular mechanisms through the identification of regulatory variants from the GWAS signals.

Recent genome-wide functional studies, such as ENCODE, FANTOM5, GTEx project, and Roadmap Epigenomics have found enormous regulatory elements across many different tissues or cell lines in the human genome. These findings provided an opportunity to glean insights into how these non-coding variants potentially affect biological functions [17]. Accordingly, algorithms that utilized such information could be applied to distinguish these putative functional variants from the GWAS loci. Several integrative algorithms have also been developed to make the results from different annotations to be comparable. For example, algorithms like Combined Annotation-Dependent Depletion (CADD) [18], Genome Wide Annotation of Variants (GWAVA) [19], and Eigen [20], which were based on machine learning approaches, could integrate many diverse annotations into a single measure for detected variant. These algorithms showed better performance than any single individual annotation, and they could be applied to get a comprehensive evaluation on the relative pathogenicity of each genetic variant.

In the current study, we performed the first integrative analyses on CCT-associated loci, including functional annotations, enrichment analyses, and protein-protein interaction analyses. Our study demonstrated the potential of data integration to identify regulatory variants in CCT GWAS findings, and also highlighted the collagen and extracellular matrix pathways in the regulation of CCT.

## Materials and methods

### Study design

**Fig. 1:**
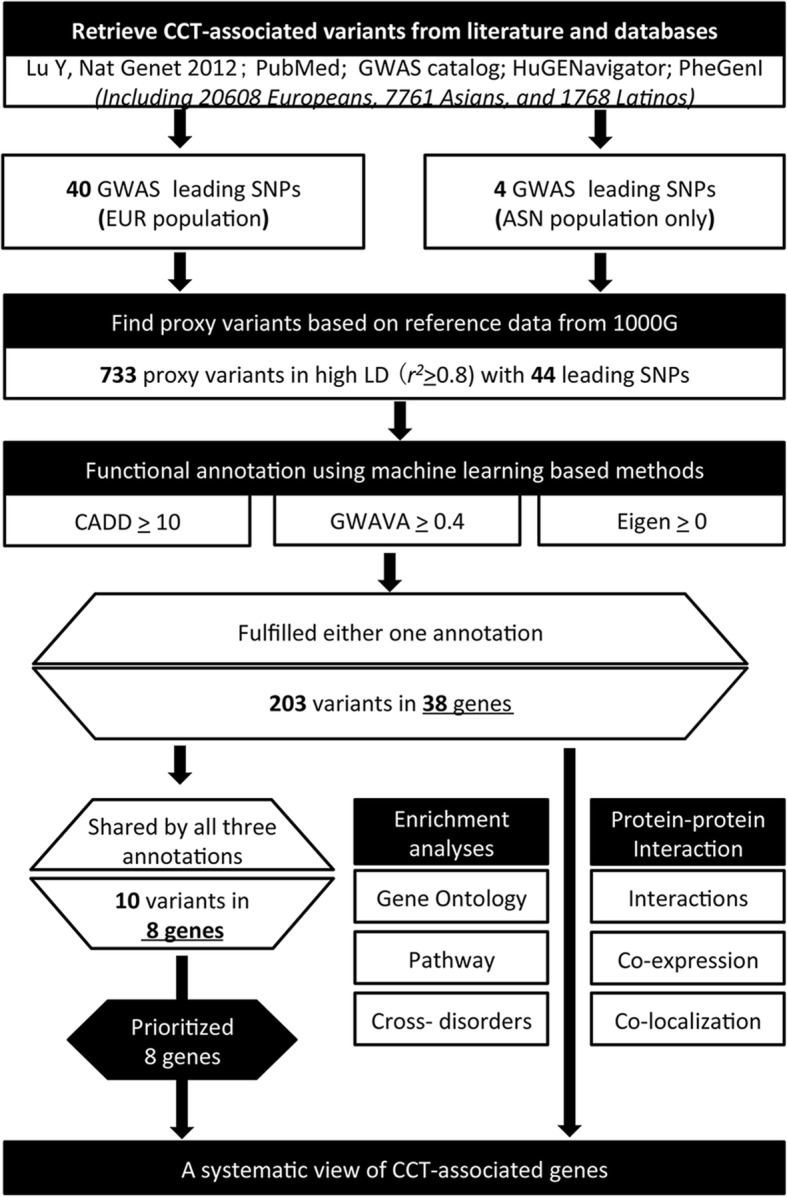
Study design of the current integrative analyses

First, CCT-associated 34 lead SNPs from Lu et al. report [9] were treated as the main dataset in the current study, and other CCT-associated variants were extracted from the following electronic databases: PubMed, GWAS catalog, HuGE GWAS integrator, and PheGenl database (accessed July 28, 2017). The reports that included in the current study were shown in Additional file 1: Table S1. After removing duplicated records, a total of 44 CCT-associated variants were selected for further analysis, and four of them showed association signals in Asian cohort only. Proxy variants that in high (LD (*r*^*2*^≥0.8) with the four Asian-specific lead SNPs were obtained from HaploReg v4 web server (accessed July 29, 2017), using reference data from ASN population of 1000 genome project, while reference data from EUR population of 1000 genome project was applied for other lead SNPs. Secondly, integrative functional annotations of the lead SNPs and their proxy variants were conducted via three algorithms, including CADD, GWAVA and Eigen score software. Variants that predicted to be functional by all the three algorithms were considered as prioritized putative regulatory SNPs, and the corresponding genes of variants that fulfilled either one of the methods were subjected to following analyses. Finally, Gene Ontology (GO) enrichment analysis together with gene co-expression network and protein-protein interaction analysis were conducted for the filtered gene set, using ToppGene Suite and GeneMANIA (a Cytoscape 3.3.0 plugin), respectively. The flowchart of the study design was illustrated in Fig. 1.

### Integrative functional annotations

Three different algorithms were used for integrative functional annotations of lead SNPs and their proxy variants.

CADD is a tool for scoring the deleteriousness of single nucleotide variants as well as insertion/deletions variants in the human genome. It integrates diverse genome annotations using conservation matrices and protein based matrices (~ 63 different tools) into a single measure (C-score) for each variant. CADD utilizes support vector machine (SVM), a supervised learning approach to contrast the annotations of fixed alleles in humans with those of simulated variants. We determined the threshold of pathogenesis as having a C-score exceeding 10, which indicated the top 10% deleterious state among possible substitutions.

GWAVA is a machine-learning algorithm (random forest) trained by the annotations from ENCODE, GECODE, and other sources to evaluate the effect of regulatory variants in noncoding regions of the genome. A normalized score of 0 to 1 is provided to reflect pathogenicity of variants, where higher scores indicate variants more likely to be functional. The TSS score of GWAVA, which incorporates various regulatory annotations, was adopted in the current study with a cutoff of 0.4.

Eigen is an unsupervised spectral approach for scoring variants without making use of labeled training data. It performs well as compared to existing methods in both coding and noncoding regions. The Eigen-PC score, which represents the first principle component of the covariance matrix, shows better performance in the prediction of noncoding variants. Variants with higher Eigen-PC scores are more likely to be functional, and a cutoff of 0 was adopted in this study.

### Gene list enrichment analysis

GO enrichment analysis, pathway analysis, and cross-disorder analysis have been performed using the ToppGene Suite, which is a one-stop portal for gene list enrichment analysis and candidate gene prioritization based on functional annotations and protein-protein interactions network. The list of CCT-associated genes was used as the input seed. Enrichment *P*-values were calculated using probability density function, and the Benjamini-Hochberg procedure was used for multiple testing corrections.

### Protein-protein interaction (PPI) and co-expression network analysis

PPI network was constructed from GeneMANIA, which searches many large, publicly available biological datasets to find related genes. GeneMANIA contains several network categories, including co-expression, physical interactions genetic interactions, shared protein domains, co-localization, pathway, and predicted functional relationships between genes. The constructed composite network was a weighted sum of individual data sources. The list of CCT-associated genes was set as the input seed.

### URLs

GWAS catalog: https://www.ebi.ac.uk/gwas/;

HuGENavigator: https://phgkb.cdc.gov/PHGKB/hNHome.action;

PheGenI: http://www.ncbi.nlm.nih.gov/gap/phegeni;

HaploReg v4.1: http://www.broadinstitute.org/mammals/haploreg/haploreg.php;

CADD: http://cadd.gs.washington.edu/;

GWAVA: https://www.sanger.ac.uk/sanger/StatGen_Gwava;

ToppGene Suite: https://toppgene.cchmc.org/;

GENECODE: http://www.gencodegenes.org/;

GTEx Portal: http://www.gtexportal.org/home/.

## Results

### Integrative functional annotations of lead SNPs and proxy variants

Figure 1 outlined our study strategy. After an initial search, a total of 44 CCT-associated lead SNPs were selected for further analysis. The proxy variants that in high LD (*r*^*2*^≥0.8) with these lead SNPs were identified, including 686 SNPs, 15 insertions and 32 deletions (Additional file 2: Table S2). Then, all the lead SNPs and the proxy variants were performed for integrative functional annotations, using three algorithms, CADD, GWAVA, and Eigen.

**Fig. 2:**
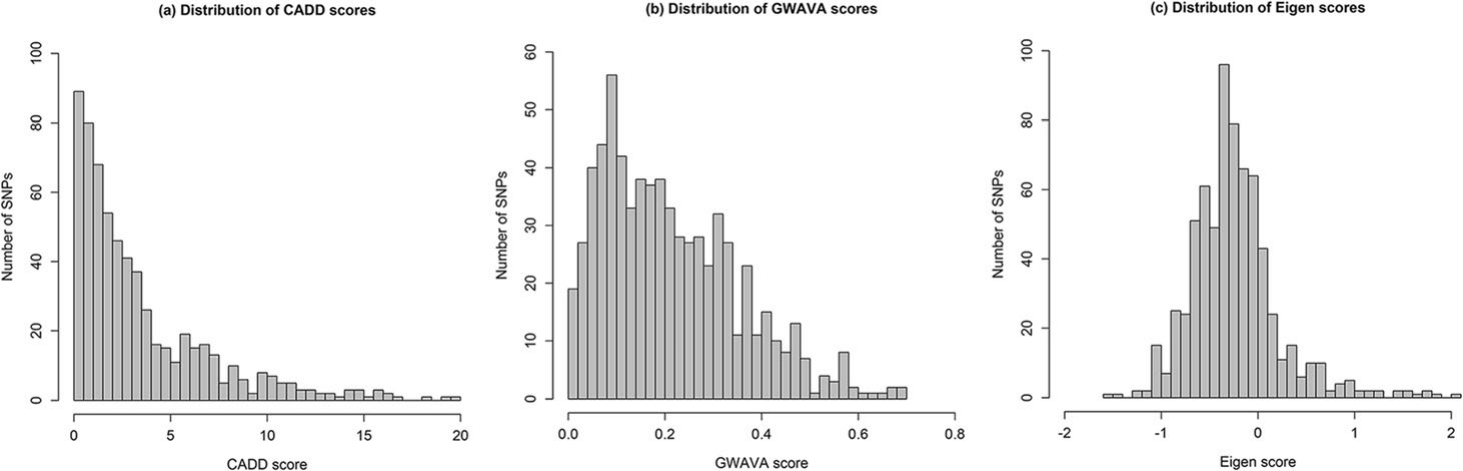
Distribution of (**a**) CADD, (**b**) GWAVA, and (**c**) Eigen scores. Histograms are drawn based on CADD, GWAVA, and Eigen scores of all the variants (both lead GWAS SNPs and LD variants) after removing the missing values

**Fig. 3:**
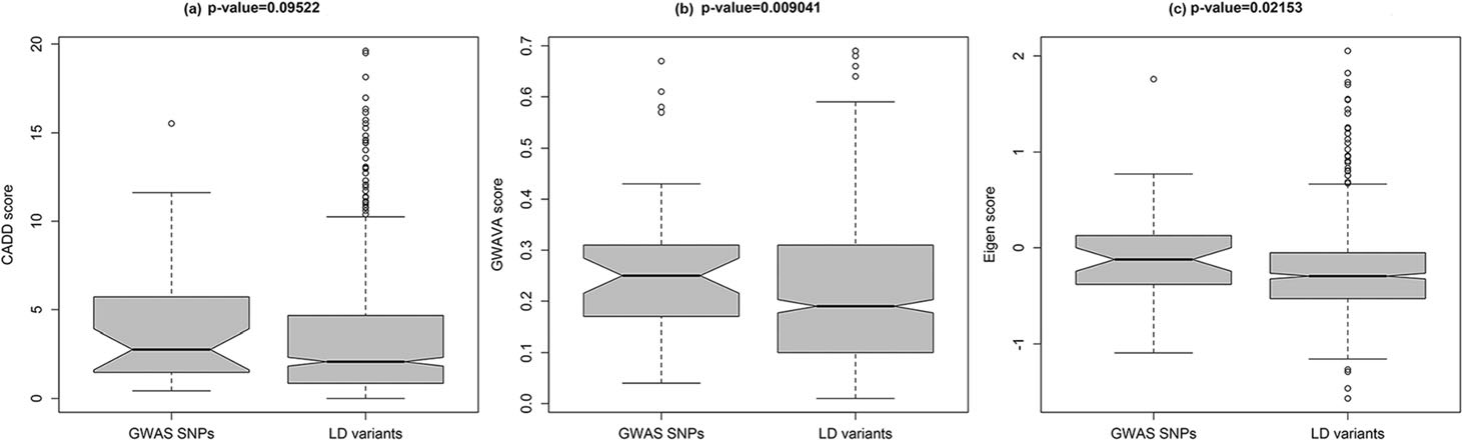
Comparison of functional annotation scores of CCT-associated GWAS SNPs versus LD variants. **a** CADD scores for GWAS SNPs (*n* = 34) versus LD variants (*n* = 587); **b** GWAVA scores for GWAS SNPs (*n* = 41) versus LD variants (*n* = 654); **c** Eigen scores for GWAS SNPs (*n* = 43) versus LD variants (*n* = 645). In the boxplots, centerlines indicated the medians of the values and box limits showed the 25^th^ &75^th^ percentiles (as determined by R package). Whiskers extended to 5^th^ and 95^th^ percentiles and outliers were shown by open circle dots. The notches were defined as ±1.58 × IQR (interquartile range)/square root of n and indicated the 95% confidence interval for each median. *P*-values were calculated using two-sided Wilcoxon rank sum test

Among the 617 variants that had C-scores based on CADD annotation, forty-four of them had C-scores exceeding 10. The distribution of C-scores for the 617 variants with available C-scores were shown in Fig. 2a, ranging from 0 to 19.6. The top five ranked variants were rs9510275, rs5814193, rs160826, rs57817160, and rs1875097, with a C-score of 19.61, 19.49, 18.13, 16.96 and 16.32, respectively. The lead SNPs had slightly higher C-scores than their corresponding proxy variants, although the *P-*value did not reach the significant level (Fig. 3a).

A total of 690 variants had TSS scores based on GWAVA annotation, and eighty-five of them had TSS scores larger than 0.4. The distribution of TSS-scores for these 690 variants was presented in Fig. 2b. The top five ranked variants were rs9409901, rs57817160, rs7170235, rs4718428 and rs9630, showing a TSS score of 0.69, 0.69, 0.68, 0.67, and 0.66, respectively. Interestingly, a significant difference in the distribution of TSS scores was found between lead SNPs and their proxy variants (*P-*value = 0.009041, two-sided Wilcoxon rank sum test, Fig. 3b).

There were 144 out of 681 variants showed positive Eigen-PC scores. The distribution of the Eigen-PC scores was shown in Fig. 2c. SNP rs2393729, rs2393730, rs2307121, rs6769466, and rs6443477 represented the top five ranked variants, with an Eigen-PC score of 2.05, 1.82, 1.76, 1.72, and 1.70, respectively. In agreement with the results from GWAVA annotation, we also observed a significant difference of the distribution of Eigen-PC scores between lead SNPs and their proxy variants (*P-*value = 0.02153, two-sided Wilcoxon rank sum test, Fig. 3b).

### Enrichment analysis

In order to reduce potential false positives, a threshold for each algorithm was adopted here (CADD≥10, GWAVA≥0.4, and Eigen-PC≥0) [21]. It was found that 10 SNPs from 8 loci showed consistent results in all three algorithms (Table 1), which were considered as prioritized putative regulatory SNPs, the corresponding genes were *ADAMSTS6, ARID5B, FOXO1, AKAP13, COL4A3, COL8A2, TBL1XR1,* and *KCMB2*. Meanwhile, we found a total of 203 variants that fulfilled either one of the three cutoff scores, and the corresponding genes of these variants were retrieved from GENECODE database. Finally, a total of 38 non-overlapped genes were subjected to the following enrichment analyses.

**Table 1:** List of prioritized putative regulatory SNPs

Variant	Chr	Position	LD (r^2^)	Lead SNP	Ref	Alt	CADD	GWAVA	Eigen-PC	Genes
rs2307121	5	65,329,685	1	rs2307121	C	T	15.53	0.57	1.76	ADAMTS6
rs2393729	10	62,077,257	0.98	rs7090871	C	T	13.08	0.41	2.05	ARID5B
rs2721051	13	40,536,747	1	rs2721051	C	T	11.62	0.58	0.65	FOXO1
rs4843047	15	85,316,545	0.83	rs6496932	G	A	10.22	0.43	0.67	AKAP13
rs57817160	2	227,314,522	0.92	rs7606754	G	A	16.96	0.69	0.91	COL4A3
rs59257065	2	227,314,612	0.91	rs7606754	C	T	15.72	0.64	0.91	COL4A3
rs6443477	3	177,590,145	0.99	rs7620503	A	C	14.6	0.57	1.70	TBL1XR1, KCMB2
rs6693322	1	36,099,761	0.8	rs96067	T	C	10.78	0.49	1.26	COL8A2
rs6769466	3	177,590,226	0.99	rs7620503	G	A	15.53	0.55	1.72	TBL1XR1, KCMB2
rs6862857	5	65,334,310	1	rs2307121	G	C	13.57	0.58	1.54	ADAMTS6

Position (hg19). Ref: Reference allele. Alt: Alternative allele. Cutoff: CADD≥10, GWAVA ≥0.4 and Eigen-PC≥ 0

In order to investigate the potential biological implication underlying this list of genes, the Gene Ontology (GO) enrichment analyses for the 38 CCT-associated genes were performed using ToppGene suite [22]. For GO term Molecular Function (MF), five terms had a false discovery rate (FDR) with B&H correction less than 0.05. The top significant terms were “transcription factor activity, protein binding” and “extracellular matrix structural constituent”. For GO term Biological Process (BP), a total of 59 terms showed FDR B&H value less than 0.05, and the most significant term was involved in connective tissue development. For GO term Cellular Component (CC), we got 10 terms having an FDR B&H < 0.05, with the top significant terms including basement membrane, extracellular matrix component, and collagen trimer. The top five GO terms were shown in Table 2, and the full list of enrichment results was shown in Additional file 3: Table S3.

**Table 2:** Top 5 terms of the Gene Ontology, Pathway, and Disease enrichment of 38 CCT-associated genes

Term	Name	*P*-value	FDR B&H
GO: Molecular Function			
GO:0000988	transcription factor activity, protein binding	1.47E-04	3.62E-02
GO:0005201	extracellular matrix structural constituent	4.54E-04	3.88E-02
GO:0033293	monocarboxylic acid binding	4.72E-04	3.88E-02
GO:0008013	beta-catenin binding	6.68E-04	4.13E-02
GO:0000989	transcription factor activity, transcription factor binding	1.01E-03	4.96E-02
GO: Biological Process			
GO:0061448	connective tissue development	6.57E-07	9.12E-04
GO:0072359	circulatory system development	3.05E-06	1.41E-03
GO:0072358	cardiovascular system development	3.05E-06	1.41E-03
GO:0001501	skeletal system development	6.12E-06	2.12E-03
GO:0048646	anatomical structure formation involved in morphogenesis	2.15E-05	5.58E-03
GO: Cellular Component			
GO:0005604	basement membrane	5.18E-05	7.82E-03
GO:0044420	extracellular matrix component	1.41E-04	1.06E-02
GO:0016327	apicolateral plasma membrane	5.81E-04	1.84E-02
GO:0043218	compact myelin	5.81E-04	1.84E-02
GO:0005581	collagen trimer	6.08E-04	1.84E-02
Pathway			
M3005	Genes encoding collagen proteins	1.41E-04	3.09E-02
1,470,926	Collagen chain trimerization	1.72E-04	3.09E-02
83,105	Pathways in cancer	2.51E-04	3.09E-02
M17668	Rho-Selective Guanine Exchange Factor AKAP13 Mediates Stress Fiber Formation	3.41E-04	3.09E-02
1,270,247	Assembly of collagen fibrils and other multimeric structures	3.56E-04	3.09E-02
Disease			
C0022578	Keratoconus	6.92E-11	8.68E-08
C1096274	Corneal thinning	1.21E-05	7.56E-03
C0544008	Chandler syndrom	6.00E-05	1.88E-02
C1857569	Corneal endothelial dystrophy 2	6.00E-05	1.88E-02
C3542501	Acute inflammatpry demyelinating polyneuropathy	1.12E-04	2.78E-02

The list of CCT-associated genes was set as the input seed. Enrichment *P*-values were calculated using probability density function, and the Benjamini-Hochberg procedure was used to correct the *P* values (FDR) of the enriched GO terms

Pathway analysis of the 38 CCT-associated genes showed that these genes were significantly enriched in 6 pathways (FDR B&H < 0.05). The top ranked pathways were mainly related to collagen and formation of extracellular matrix structure, as curated by KEGG, REACTOME and BioCarta database (Table 2 and Additional file 3: Table S3).

Cross-disorder analysis showed the most significant enrichment of these CCT-associated genes with the phenotype of keratoconus (adjusted enrichment *P*-value = 8.68E-08, Table 2 and Additional file 3: Table S3). We then evaluated our prioritization scheme by comparing the enrichment scores between each individual prediction tool and the combined approach. We found that all the enrichment scores for keratoconus were statistically significant with any single prediction tool, while there was a substantial increase in the enrichment scores when the combined approach was applied (Table 3).

**Table 3:** Summary of the enrichment scores for genes prioritized for keratoconus (KC) using a combination of CADD, GWAVA, and Eigen prediction tool

Prediction tool	Fold Enrichment	FDR B&H
CADD	32.2	9.54E-03
GWAVA	23.9	8.17E-03
Eigen-PC	20.1	2.14E-02
CADD+GWAVA+Eigen-PC	77.9	8.68E-08

Fold-enrichment was calculated as ((m/n)/(M/N), where m is the number of prioritized genes (with a tool or a combination of tools) belong to KC, n is the total number of genes prioritized using a tool (or a combination of tools), M is the total number of genes associated with KC, and N is the human genome background (around 20,000 genes). Enrichment *P*-values were calculated using probability density function, and the Benjamini-Hochberg procedure was used for multiple testing corrections

### Interaction network

**Fig. 4:**
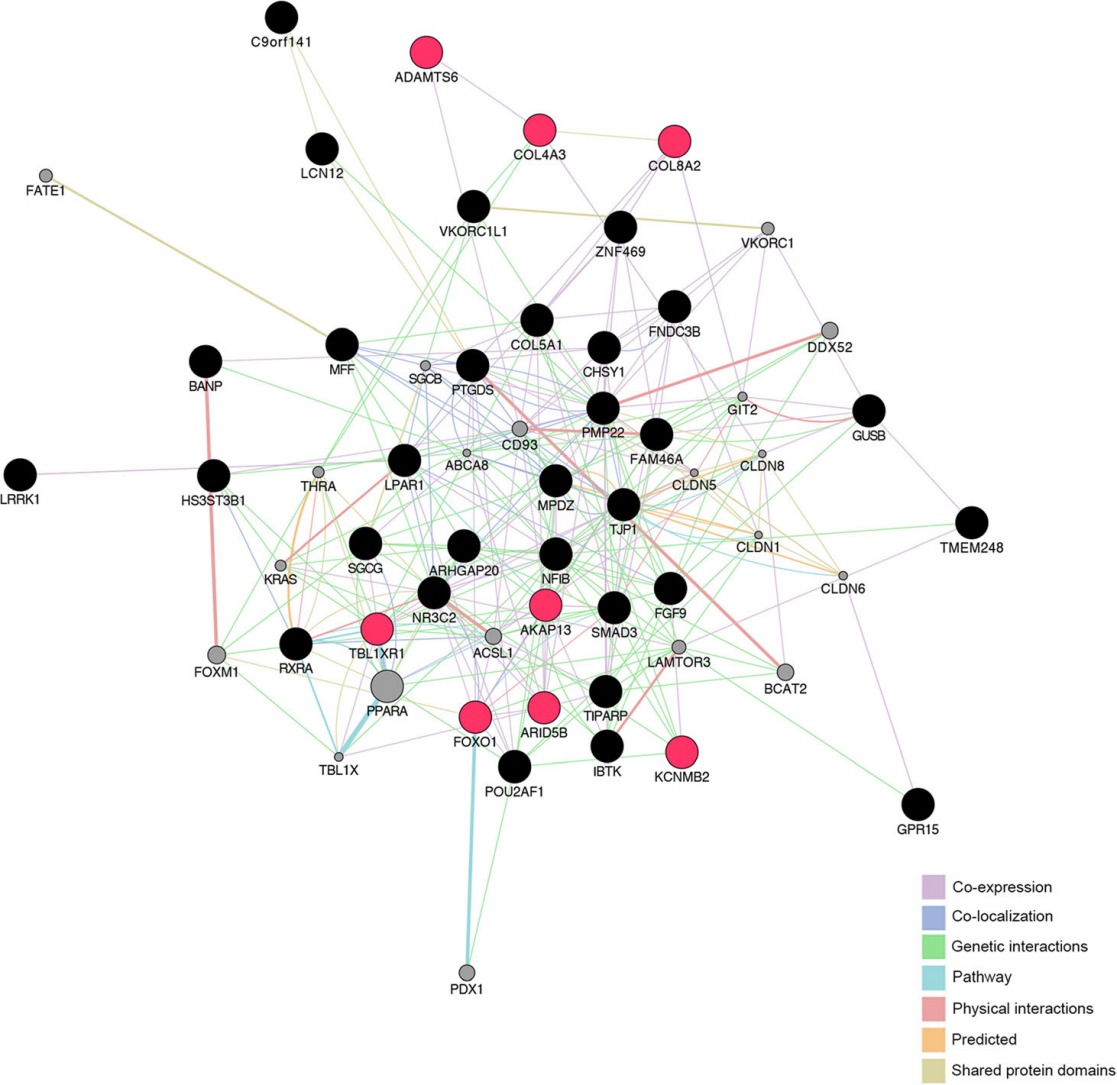
Protein-protein interaction (PPI), co-expression network analyses using GeneMANIA. Genes were shown as nodes and edges indicated different types of interaction among genes. Black circles were the query genes and the colors coding on edges indicated different types of interaction. The eight prioritized genes were labeled with red circles

Protein-protein interaction network and gene co-expression analyses were performed to investigate the potential relationship among the 38 CCT-associated genes. As shown in Fig. 4, these genes were involved in one interaction network. *RXRA* and *TBL1XR1*, *LPAR1* and *AKAP13* were found to be involved in the same pathways, while *FOXO1* and *SMAD3* showed direct interaction with each other. For the eight genes that have been prioritized by three integrative functional annotations, different patterns of interaction have been found between them and several other CCT-associated genes or potentially linked genes.

## Discussion

In this study, by performing integrative analyses of publically available datasets, we have intensively explored the possible functional relevance of CCT-associated loci. A number of hub genes were found to be in the center of CCT genetic network. This study provided a new insight into the genetic regulation underlying CCT GWAS findings, and also indicated that integrative analysis might be an important supplementary method for exploring functional mechanisms underlying CCT GWAS findings.

Based on the combined functional annotations, most of the CCT-associated genes were significantly involved in the metabolic activities associated with collagen and extracellular matrix, which was consistent with previous studies. In addition, we prioritized eight genes (*ADAMSTS6, ARID5B, FOXO1, AKAP13, COL4A3, COL8A2, TBL1XR1,* and *KCMB2*) harboring SNPs with strong evidence of regulatory potential. Most notably, rs4843047 in *AKAP13*, rs57817160 and rs59257065 in *COL4A3*, and rs6693322 in *COL8A2* have also been confirmed as eQTLs by the GTEx Portal, implying their potentially direct roles in affecting expression of the corresponding genes. These prioritized genes might be potential candidates to investigate the genetic predisposition to some particular ocular abnormalities like keratoconus or POAG that mediated through the genes underlying CCT. Indeed, the contribution of variant near *FOXO1* to keratoconus susceptibility has already been identified by Lu’s study [9]. However, the role of other genes has not been validated yet. The underlying reasons may include the limited sample size in the original studies; the incomplete LD between these prioritized putative regulatory SNPs and the interrogated SNPs in the original studies; or the potential heterogeneity across different populations. Therefore, further investigations on these putative regulatory SNPs might also be valuable when studying the genetics of CCT-associated diseases.

Cross-phenotype analysis did not yield unexpected predictions, while these CCT-associated genes were significant enriched in phenotype of keratoconus and corneal thinning. Interestingly, direct interaction between *SMAD3* and *FOXO1* was found by PPI analysis. *FOXO1* is a well-established susceptibility gene for keratoconus, while the association of *SMAD3* with keratoconus susceptibility remains unclear. Although a SNP in *SMAD3* only showed nominal significance in association with keratoconus in the same study [9], it is possible that other SNPs might confer the risk, and further fine mapping approach is required to get the whole picture of this region.

The study conducted by Lu [9] included 13 GWAS on CCT, totaling over 20,000 individuals with European or Asian ancestry. Their observations showed that most of the CCT-associated loci identified from populations of European were shared in Asian populations, suggesting that the underlying genetic effects were largely shared between the two ancestry groups. However, some potential heterogeneity still existed within particular locus. For example, except for the consistent association of SNP rs1536482 in the *RXRA-COL5A1* locus between Europeans and Asians, another independent SNP in the same locus, rs1536478 was found to be associated with CCT only in Asians. The same scenario was also observed in the *LRRK1* and *AKAP13* loci, as SNP rs4963359, rs1828481, and rs7172789 showed significant signals only in Asians as well.

For variant annotation, a combined approach containing three different algorithms was applied in this study. CADD and GWAVA were two supervised machine-learning algorithms, which were based on SVM and random forest method, respectively. They have shown excellent performance in estimating the relative pathogenicity of human genetic variants, and have been successfully applied in identifying the regulatory variants that conferring risks for inflammatory bowel disease [21] and several psychiatric diseases [23]. Eigen was an unsupervised machine learning method. This algorithm did not rely on any labeled training data, so it could reduce the dependence on existing databases of observed variants, previously characterized elements, and existing models of mutation, a scenario that usually encountered when limited information about the disease/trait was available [20]. We also evaluated the performance between the combined approach and each individual annotation by calculating the disease enrichment scores of different strategies. Our analysis showed that a combined approach could be very effective to annotate and prioritize putative functional noncoding variants from GWAS loci.

In summary, through comprehensive integrative analyses, the current study revealed the hub genes that were located in the center of CCT genetic network, and provided a new insight into the genetic regulation underlying CCT GWAS findings. However, several limitations existed in this study. Although some variants with probable regulatory functions were identified from the established GWAS loci, we could not be conclusive for their association since none of these prediction algorithms were disease specific. In addition, conclusions revealed by our integrative analyses were heavily dependent on limited number of reported CCT-associated genes, for other immune-related diseases like systemic lupus erythematous, type 1 diabetes and inflammatory bowel disease, with much more susceptible variants have been identified, integrative analyses showed better performance in revealing their functional relevance for the diseases [21, 24, 25]. Therefore, the overall profile revealed by this study requires further validation with larger sample size in the future.

## Additional files

Additional file 1: **Table S1.** GWAS reports that included in the current study. (XLSX 39 kb)

Additional file 2: **Table S2.** Summary of integrative annotation scores of CCT associated lead SNPs and variants in strong linkage-disequilibrium (LD) (r2 ≥ 0.8) with the lead SNPs, using CADD, GWAVA and Eigen tools. (XLSX 118 kb)

Additional file 3: **Table S3.** Significant terms of the Gene Ontology, Pathway, and Disease enrichment of 38 CCT-associated genes (FDR < 0.05). (XLSX 55 kb)
